# Host-plant genotypic diversity and community genetic interactions mediate aphid spatial distribution

**DOI:** 10.1002/ece3.916

**Published:** 2013-12-15

**Authors:** Sharon E Zytynska, Laurent Frantz, Ben Hurst, Andrew Johnson, Richard F Preziosi, Jennifer K Rowntree

**Affiliations:** Faculty of Life Sciences, University of ManchesterMichael Smith Building, Manchester, M13 9PT, U.K

**Keywords:** Barley, multitrophic, parasitic plant, performance, phytophagous insect, plant–insect, preference.

## Abstract

Genetic variation in plants can influence the community structure of associated species, through both direct and indirect interactions. Herbivorous insects are known to feed on a restricted range of plants, and herbivore preference and performance can vary among host plants within a species due to genetically based traits of the plant (e.g., defensive compounds). In a natural system, we expect to find genetic variation within both plant and herbivore communities and we expect this variation to influence species interactions. Using a three-species plant-aphid model system, we investigated the effect of genetic diversity on genetic interactions among the community members. Our system involved a host plant (*Hordeum vulgare*) that was shared by an aphid (*Sitobion avenae*) and a hemi-parasitic plant (*Rhinanthus minor*). We showed that aphids cluster more tightly in a genetically diverse host-plant community than in a genetic monoculture, with host-plant genetic diversity explaining up to 24% of the variation in aphid distribution. This is driven by differing preferences of the aphids to the different plant genotypes and their resulting performance on these plants. Within the two host-plant diversity levels, aphid spatial distribution was influenced by an interaction among the aphid's own genotype, the genotype of a competing aphid, the origin of the parasitic plant population, and the host-plant genotype. Thus, the overall outcome involves both direct (i.e., host plant to aphid) and indirect (i.e., parasitic plant to aphid) interactions across all these species. These results show that a complex genetic environment influences the distribution of herbivores among host plants. Thus, in genetically diverse systems, interspecific genetic interactions between the host plant and herbivore can influence the population dynamics of the system and could also structure local communities. We suggest that direct and indirect genotypic interactions among species can influence community structure and processes.

## Introduction

Genetic variation within a species is the basis for evolutionary change in a population, and different genotypes within a species can show variation in their response to different environments (Agrawal 2001). Such environments can arise through the presence or absence of other species in a community, which interact with the focal species through, for example, competition or predation. Community genetics research has shown that within-species genetic variation can change the magnitude and direction of the outcome of direct and indirect interactions among species (Service 1984; Carius et al. 2001; Tetard-Jones et al. 2007; Zytynska et al. 2010; Rowntree et al. 2011a). In other words, the genotypes of the individuals interacting are important for the outcome in a multispecies community.

In a genetically diverse system, insect preference for certain host plants will influence the distribution of insects in a population and can occur through both feeding and oviposition site choices. These choices can have ecological and evolutionary consequences, with host-associated differentiation potentially driving ecological speciation (Stireman et al. 2005; Matsubayashi et al. 2009). Sap-sucking insects, such as aphids, experience an intimate relationship with their host plant and many aphid species exhibit genetic variation in host preference and performance on different host plants (Via 1991; Debarro et al. 1995; Nikolakakis et al. 2003; Ferrari et al. 2006; Gorur et al. 2007). At the plant genetic level, different aphid genotypes have also been found to preferentially colonize different host-plant genotypes (Zytynska and Preziosi 2011), which means that a population can be spatially structured through indirect genetic effects (IGEs). IGEs occur when the phenotype of one individual changes due to the expressed genes in another interacting individual (Wolf et al. 1998), and are known as IIGEs (interspecific indirect genetic effects) when they occur across species (Astles et al. 2005; Shuster et al. 2006; Whitham et al. 2006). In a genetically and species-diverse environment, IIGEs can also be modified due to the presence of other interacting species (Tetard-Jones et al. 2007; Zytynska et al. 2010). Therefore, the spatial distribution of aphids among host plants in a community may be determined by plant-aphid IIGEs on host preference and performance. In addition, the species composition of the interacting community (i.e., other plants, predators, or soil organisms) could also mediate the genetic effect of the host plant on the aphids leading to community-wide effects on their distribution. It has been found in plant–insect systems that insects do not always choose to feed or reproduce on the plants that infer the highest fitness (Thompson 1988). In a diverse system, with a high number of interacting individuals and species, the optimal host plant within a population may change over time as the interacting community alters and changes the IIGEs. For example, changes in optimal host-plant individual might be due to genetically based variation in nutrition over the growing season (Stamp and Bowers 1990), induced defenses (Soler et al. 2012; Bernhardsson et al. 2013) or as a response to direct and indirect species interactions (Agrawal et al. 2012; Genung et al. 2013).

In this article, we use a three-species plant–insect system to look at the effect of genetic variation in each species on the spatial distribution of aphids on their host plants. This system contains aphids (*Sitobion avenae*) that feed on a host plant (*Hordeum vulgare*; barley), and a hemi-parasitic plant (*Rhinanthus minor*) that parasitizes the barley, but is not a suitable host for the aphids. Different aspects of the system have been previously studied, which provides us with information regarding specific interactions among the species. However, these species have not previously been combined into a single experimental system. We know that interactions between *S. avenae* and barley are affected by the abiotic environment, through changes in the soil nutrient levels (Rowntree et al. 2010) and by the biotic environment, through the presence of soil rhizobacteria around the barley root system (Tetard-Jones et al. 2007). Further work has shown that these genotypic interactions between the aphid and plant mediate the effect of soil bacteria on the body size of an aphid parasitoid wasp (Zytynska et al. 2010) and can influence plant–aphid preference/performance relationships (Zytynska and Preziosi 2011). These plant–aphid preference/performance relationships are also mediated by competition among the different aphid genotypes (Zytynska and Preziosi 2013). All aphids tested by Zytynska and Preziosi (2011) showed a preference against one particular barley plant (OWBrec), but preferences for host plants were more aphid-genotype specific. In the absence of aphids, genetic variation within barley and *R. minor* is also known to affect the outcome of interactions between these two plant species, such that the virulence and fitness of the parasite, depends on the genetics of both the host and parasite (Rowntree et al. 2011a).

The majority of previous work on this system has thus focused on genotypic interactions between the aphids and the plants, when maintained in predominantly single genotype combinations. Here, we use three species in the system and incorporate genetic variability at all levels. We aim to determine how genotypic diversity of the host plant, genetic variation of the hemi-parasitic plant, and genetic variation among the aphids interact to alter the spatial distribution of aphids within the system. We hypothesize that aphids will be more evenly distributed among plants in a low-diversity system than in a high-diversity system due to reduced effects of preference, performance, and species interactions.

## Materials and Methods

### Model system and experimental design

**Figure 1:**
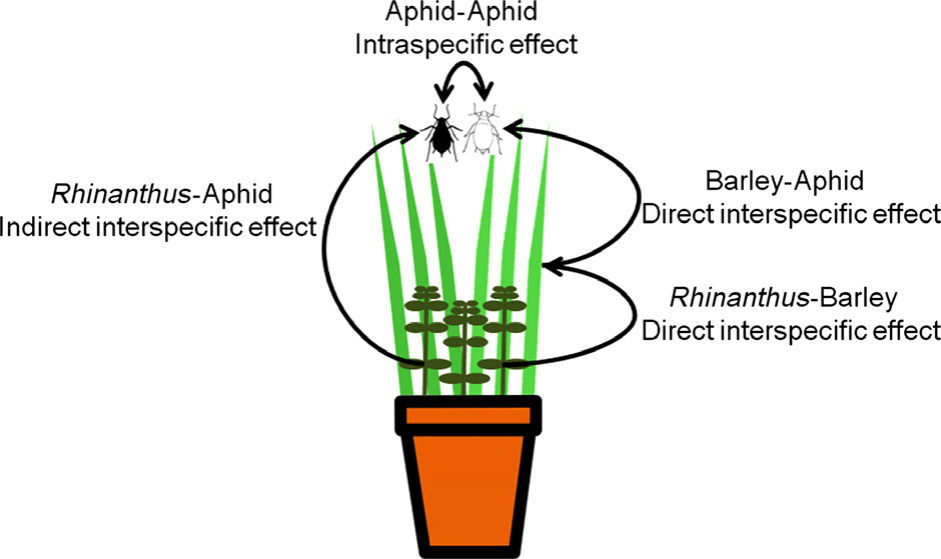
We used a system involving barley, *Rhinanthus,* and aphids to test the effects of inter-and intraspecific genetic interactions on aphid number and their spatial distribution. We had two host-plant diversity levels (genetically uniform and genetically diverse barley), three hemi-parasitic *Rhinanthus* treatments (absent, two populations) and each pot contained two aphid color morphs (multiple genotypes) to test intraspecific effects. We calculated how much aphids clustered in the pots to see whether genetic interactions among a multispecies community can influence aphid distribution.

Our three-species model system consisted of a host plant (barley; *Hordeum vulgare* L.) that was shared by an aphid, *Sitobion avenae*, and a hemi-parasitic plant, *Rhinanthus minor* L. (Fig. 1). For the experiment, we used six double haploid barley genotypes (Morex, Steptoe, Blenheim, Kym, OWBrec, and OWBdom) originally obtained from P. Hayes (Oregon State University, USA); two populations of *Rhinanthus*: “Inverness” obtained from Scotia Seeds (Brechin, Angus, UK) and “Somerset” from Emorsgate Seeds (Kings Lynn, Norfolk, UK); and four aphid genotypes (CLO7, DAV95, H1, and HF92a) originally obtained from Rothamsted Research, Harpenden, UK, where they were identified as separate clones using microsatellites. Asexual clonal aphid populations were grown on barley genotype “pearl”. The aphids belong to two color morphs: CLO7 and HF92a are brown, and H1 and DAV95 are green. These color morphs differ in a number of traits including reproductive potential, with genotypes within the color morphs producing more similar patterns (Zytynska and Preziosi 2011) and endosymbiont infection; the green aphids possess the endosymbiont *Regiella insecticola,* and the brown aphids do not (J. Ferrari, pers. comm.).

We used a factorial experimental design. First, we used two host-plant diversity treatments: low diversity, which had six plants of a single genotype per pot with two repeats for barley genotypes Morex, Steptoe, Blenheim, and Kym, and three repeats for genotypes OWBrec and OWBdom, giving a total of 14 replicates; and high diversity, which had six plants of each of the different genotypes per pot with 12 replicates. Second, we used three *Rhinanthus* treatments (two populations plus a no *Rhinanthus* control).Third, we used four aphid treatments with one green and one brown morph always paired together (i.e., DAV95+CLO7, DAV95+HF92a, H1+CLO7, and H1+HF92a). In total, we had 24 treatments each with 12–14 starting replicates. For conciseness, we will now refer to the high-diversity host-plant treatment as HD and the low-diversity host-plant treatment as LD. Over the course of the experiment, 34 pots were removed due to host-plant death or unsuccessful attachment of *Rhinanthus* to the barley. The final number of pots was 277, with 8–12 replicates per treatment in the HD and 8–14 in LD. All treatments were randomly assigned to pots, and the different host-plant genotypes in the HD were planted in a random order such that no two genotypes were consistently located next to each other.

### Experimental set-up

The barley seeds were germinated between two moistened pieces of filter paper in petri dishes in a dark growth cabinet at 23°C for 6 days. Seedlings were transplanted into experimental pots (15 cm diameter) filled with horticultural sand, at equal spacing in a circle 2 cm from the pot edge. The *Rhinanthus* seeds were surface sterilized using 1% v/v sodium hypochlorite solution for 3 min and then germinated in the dark at 4°C over a 3–4 month period in sealed petri dishes (9 cm) containing moist, sterile filter paper, and capillary matting. The germinated seeds were transplanted into the experimental pots at the same time as the barley seedlings. Six *Rhinanthus* seedlings with approximately 1–2 cm radicles were planted in a circle approximately 2 cm toward the center from the barley plants. Once attachment of three *Rhinanthus* plants was observed, the remaining plants were removed to leave only three attached in each experimental pot. Attachment was noted through plant traits such as inflated leaves, rapid growth, and a change in leaf color from dark green to yellowish green (Klaren and Janssen 1978).

The experimental pots were placed on upturned saucers on benches in a glasshouse (temperature range 15–25°C; 16:8 photoperiod) and watered daily with 50 ml or 100 ml of 25% Hoagland's nutrient solution (Hoagland and Arnon 1950) for the duration of the experiment. The volume of nutrient solution differed over time, due to the requirements of the plants but was consistent for all pots on any 1 day. Additional water was added if pots remained dry. Six weeks after planting, we added six-fourth instar, or adult, aphids from each genotype in the aphid pairs resulting in 12 aphids per pot. These were introduced to the pots by collecting all the aphids into a 3-cm-diameter petri dish and then placing the dish in the center of the pot, to minimize bias toward any single plant. Each pot was then covered by a fine-mesh bag supported by a frame (Insectopia; Austrey, Warwickshire, UK) to ensure no aphid movement between pots. The aphids were free to move among the plants within a pot. No aphids were found on the *Rhinanthus* plants. After 2 weeks, the number of aphids on each host plant, in every pot, was counted.

### Data analysis

To analyze the spatial distribution (clustering) of the aphids in the pot, we calculated the deviation from equal distribution, that is (O−E)^2^, where O is the observed number of aphids on a plant, and E is the expected number of aphids if distribution was equal (i.e., one-sixth of the total number of aphids in the pot). An equal distribution of aphids in a pot will result in a deviation of zero. It is not expected that the distribution of aphids within a control pot (i.e., one plant genotype and no *Rhinanthus*) will be exactly equally distributed as aphids reproduce asexually, producing a cluster of young in one area before moving away to produce another cluster. However, here, we analyze the variation in the deviation, or the degree of clustering, among the plants to determine whether the experimental factors can explain significant amounts of the variation in the dataset. All statistical analyses were performed in R v3.0.1 (R Core Team 2013) using R-studio v 0.97.314 (RStudio 2012). Tables of statistical results are available from the supplementary data.

To analyze the data, we first asked whether there was a difference in aphid number and clustering between the LD and HD treatments. We did this at the pot level using the sum of deviations across all six plants in the pots. We also used plant biomass as a covariate in all models but we found no effect; therefore, it is not included in any final analyses. At the pot level, aphid number and aphid clustering (both satisfied normal error distribution assumption) were analyzed using ANOVA/ANCOVA. For our first models, our response variables were total aphid number and aphid clustering (of all aphids, summed by pot), and the independent variables were (1) host-plant genotypic diversity, (2) *Rhinanthus* treatment, and (3) aphid pair, with plant biomass as a covariate. Full models were fitted first and then simplified using the backward stepwise method and comparing fitted models using ANOVA function in R (Crawley 2012). We present the minimal adequate models and any factorial simplification (Crawley 2012) in the results. Factorial simplification is used to group levels within a factor and to determine which are driving the effect seen in the model. This method also frees up degrees of freedom thereby allowing a simplified model with a higher statistical power to be run. Our next analyses considered the number and clustering of brown and green aphids separately, still at the pot level, with our response variables of aphid clustering (two models were run, one for the green and one for the brown aphids), and the independent variables were (1) host-plant genotypic diversity, (2) *Rhinanthus* treatment, (3) focal aphid genotype, and (4) interacting aphid genotype. As every pot had two aphid genotypes, we were not able to analyze all data together as this would cause pseudoreplication in the data with increased residual degrees of freedom (hence the separation when considering intraspecific aphid genotype interactions). Next, we analyzed the data separately for the HD and LD pots. Here, we are able to include plant genotype into the models and use a linear mixed model with pot as a random factorial effect to control for pseudoreplication as there are six observations per pot; plant biomass and aphid number (for the clustering models) were also used as covariates. For these analyses, aphid clustering data were transformed (raised to power of 0.2) to achieve normal errors. Here, the response variables were total numbers of aphids (natural-log transformed to achieve normal errors), clustering of the green or brown aphid genotypes (two models were run, one for the green and one for the brown aphids), and the independent fixed effect variables were (1) host-plant genotype, (2) *Rhinanthus* treatment, (3) focal aphid genotype, and (4) interacting aphid genotype. We also ran a model for the relative number of aphids per plant in HD compared to LD pots (calculated using individual HD values each minus the mean in the LD pots for that particular treatment), with host-plant genotype, *Rhinanthus* treatment, and focal aphid genotype.

## Results

### Host-plant genotypic diversity

The total number of aphids in the pots was highly dependent on the aphid pairing (*F*_3,273_ = 9.84, *P *<* *0.001); pairs with CLO7 aphids had higher population sizes than pairs with HF92a (factorial simplification: *F*_3,275_ = 26.55, *P *<* *0.001). There was no significant effect on the total number of aphids within the pots of plant genotypic diversity (*F*_1,272_ = 3.23, *P *=* *0.069) or *Rhinanthus* treatment (*F*_1,270_ = 1.06, *P *=* *0.347). We measured the amount of aphid clustering in the pots by calculating the deviation from an even distribution across the plants. Hence, if the aphids were found to congregate on only one or two plants in a pot then the amount of clustering would be increased. Overall, there was more clustering of aphids within the HD pots compared to LD pots (*F*_1,272_ = 50.74, *P *<* *0.001), and when *Rhinanthus* was present, clustering was increased compared to when *Rhinanthus* was absent (*F*_2,272_ = 4.33, *P *=* *0.014).

In every pot, there was one green (DAV95 or H1) and one brown (CLO7 or HF92a) aphid genotype, and to avoid issues of pseudoreplication, we considered the effects on the clustering and number of each color morph separately (Table 1). Both the green and the brown aphids were more clustered in the HD pots than the LD pots (green aphids *F*_1,274_ = 47.65, *P *<* *0.001; brown aphids *F*_1,274_ = 27.45, *P *<* *0.001), with host-plant genotypic diversity explaining 24% and 16% of the variation in green and brown aphids, respectively. However, the number of aphids did not differ between the HD and LD pots (green aphids *F*_1,273_ = 2.96, *P *=* *0.086; brown aphids *F*_1,273_ = 1.86, *P *=* *0.173), indicating that the effects are due to aphid movement rather than an overall increase in performance at the pot level. We found an effect of apparent competition among the aphids, but only for the number of green aphids (Table1). *Rhinanthus* treatment did not explain any clustering of the brown aphids (Table 1), but there was a trend for *Rhinanthus* to influence the green aphid clustering (*F*_1,272_ = 2.66, *P *=* *0.072). After factorial simplification, we found that the green aphids clustered more when *Rhinanthus* was present compared to when *Rhinanthus* was absent (*F*_1,273_ = 5.23, *P *=* *0.023). Lastly, there was variation among green genotypes with DAV95 clustering more than H1 aphids (*F*_1,274_ = 6.38, *P *=* *0.012) likely driven by their higher reproductive performance (*F*_1,274_ = 12.98, *P *<* *0.001).

**Table 1:** Summary of effects of plant genotypic diversity, *Rhinanthus,* and aphid genotype on the number and clustering of the aphids at the pot level

	Brown number			Brown clustering			Green number			Green clustering		
Aphids by pot	df	F	*P*	df	F	*P*	df	F	*P*	df	F	*P*
Plant biomass	**1.274**	**4.70**	**0.031**	**1.274**	**4.69**	**0.031**	1.252	0.03	0.859	1.252	1.98	0.161
Diversity	1.273	1.86	0.173	**1.274**	**27.45**	**<0.001**	1.273	2.96	0.086	**1.274**	**47.65**	**<0.001**
Green aphid	1.272	0.19	0.669	1.272	0.26	0.613	**1.274**	**12.98**	**<0.001**	**1.274**	**6.38**	**0.012**
Brown aphid	**1.274**	**34.90**	**<0.001**	1.273	1.87	0.172	**1.274**	**4.24**	**0.041**	1.271	0.40	0.527
Rhinanthus	2.270	0.19	0.823	1.270	0.38	0.683	2.271	0.37	0.691	2.272	2.66	0.072

The values in bold were retained in the minimal adequate model. Interaction terms are not shown but when tested were found to be nonsignificant and thus removed from the model.

### Low genotypic diversity plant pots

**Figure 2:**
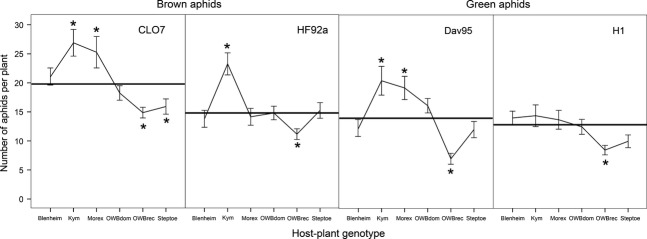
The effect of host-plant genotype on the performance of the different aphid clones in LD pots where six plants of the same host-plant genotype were planted per pot. The data are the mean number of aphids per plant across the host-plant genotypes. Thick horizontal bars show the mean aphid number per plant across all host-plant genotypes. Stars indicate where the aphid number for that host-plant genotype was significantly different (*P *<* *0.05) from the overall mean across all plant genotypes. Error bars are ±1 SE.

**Figure 3:**
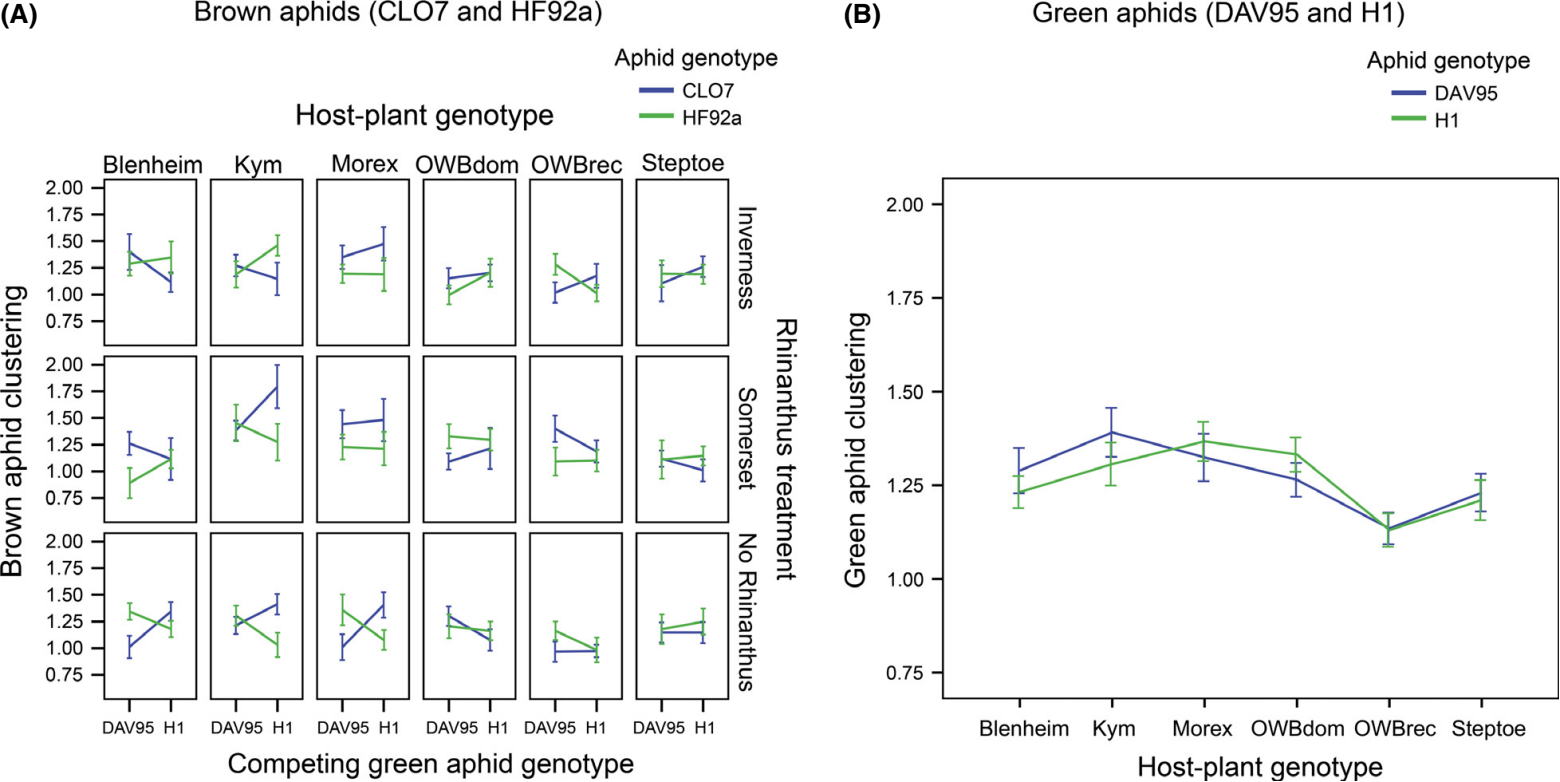
Aphid clustering in the low-diversity LD pots where six plants of the same host-plant genotype were planted per pot. We showed that (A) the clustering of the brown aphids was influenced by a complex four-way interaction at both diversity levels and (B) distribution of green aphids was influenced only by host-plant genotype. Error bars are ±1 SE.

Here, there were six plants of the same genotype, and from this, we can get an estimate of the aphid performance on the different host-plant genotypes (Fig. 2). Both the total number of brown aphids and the clustering of the brown aphids were found to be influenced by a complex four-way interaction among host-plant genotype, *Rhinanthus* treatment, its own genotype, and the genotype of the competing green aphid (aphid number: *Χ*^2^ = 20.23, df = 10, *P *=* *0.027; aphid clustering: *Χ*^2^ = 25.19, df = 10, *P *=* *0.005; Fig. 3A). The clustering of the green aphids was only influenced by host-plant genotype (*Χ*^2^ = 20.34, df = 5, *P *=* *0.001; Fig. 3B), with aphids more evenly distributed among the six host plants in pots with six OWBrec or six Steptoe plants (*t*_145_ = 4.03, *P *<* *0.001), whereas the number of green aphids was also influenced by the four-way interaction among all participants (*Χ*^2^ = 32.47, df = 10, *P *<* *0.001).

### High genotypic diversity plant pots

**Figure 4:**
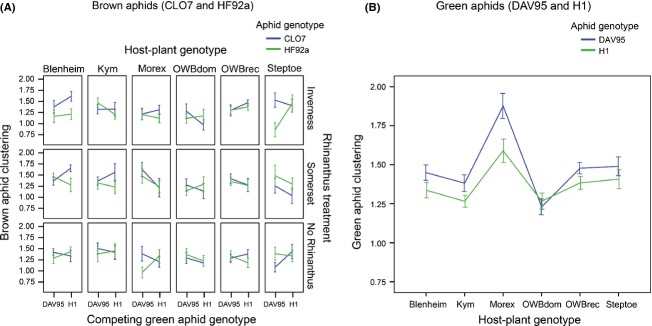
Aphid clustering in the high-diversity HD pots. Here, the six plants per pot were all from different host-plant genotypes. We showed that (A) the clustering of the brown aphids was influenced by a complex four-way interaction at both diversity levels and (B) distribution of green aphids was influenced by host-plant genotype and its own genotype. Error bars are ±1 SE.

Here, each pot contained six plants with one plant per genotype. The number and clustering of the brown aphids were again influenced by a complex four-way interaction term among host-plant genotype, *Rhinanthus* treatment, its own genotype, and the genotype of the competing green aphid (aphid number: *Χ*^2^ = 18.27, df = 10, *P *=* *0.051; aphid clustering: *Χ*^2^ = 22.72, df = 10, *P *=* *0.012; Fig. 4A). The clustering of the green aphids was influenced by their own genotype (*Χ*^2^ = 5.75, df = 1, *P *=* *0.016; Fig. 4B), with DAV95 aphids clustering more than the H1 aphids. Host-plant genotype also influenced the number of green aphids (*Χ*^2^ = 219.49, df = 5, *P *<* *0.001) and clustering of the green aphids (Χ^2^ = 67.46, df = 5, *P *<* *0.001), with aphids found more often on Morex plants (*t*_128_ = 5.85, *P *<* *0.001) and less often on OWBdom plants (*t*_128_ = 4.34, *P *<* *0.001). The *Rhinanthus* treatment and genotype of the competing aphid did not influence the clustering of the green aphids (*Rhinanthus*:*Χ*^2^ = 1.90, df = 2, *P *=* *0.387; competing aphid: *Χ*^2^ = 1.90, df = 1, *P *=* *0.168).

### Preference and performance

**Figure 5:**
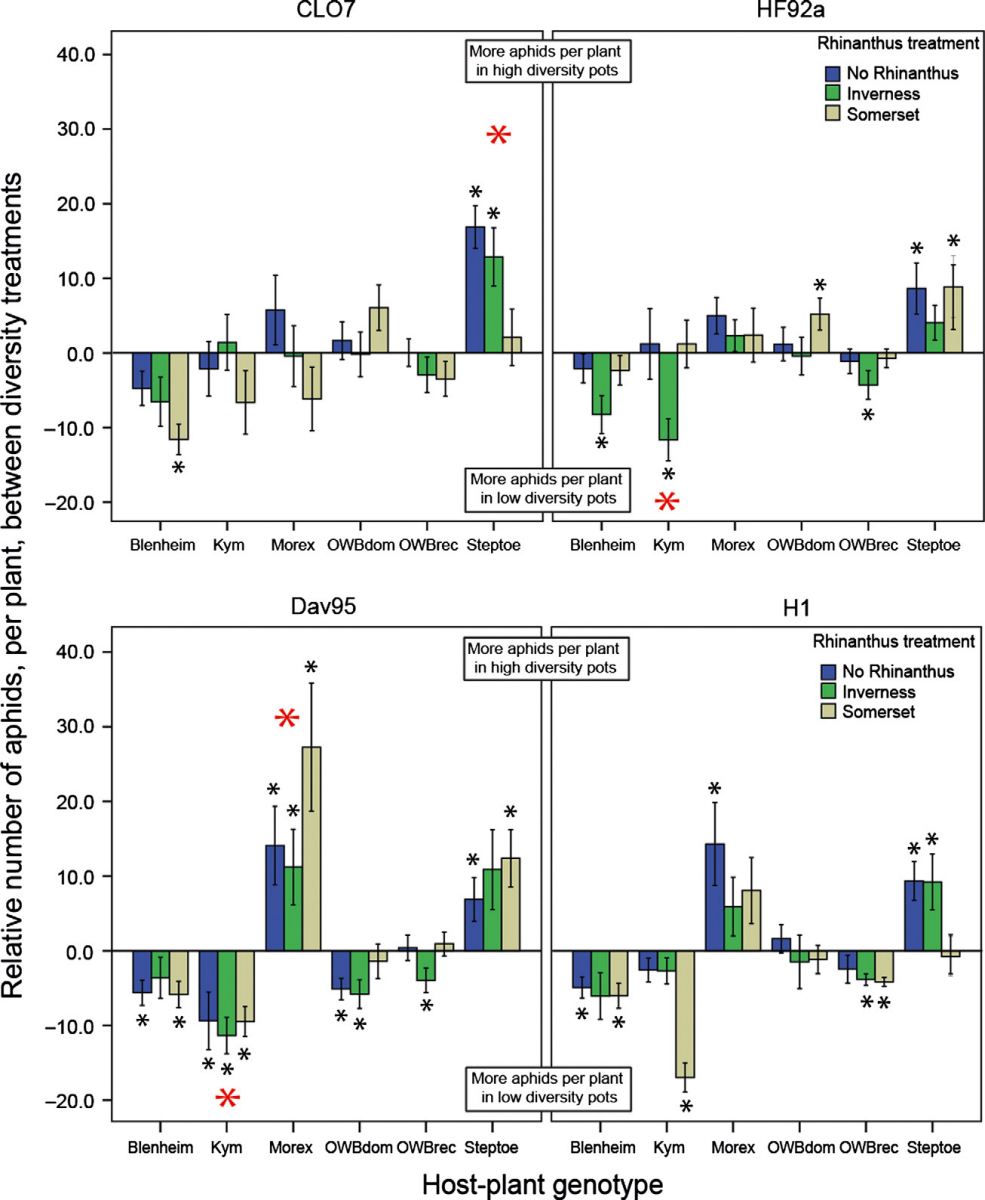
Comparison of aphid number per plant in HD and LD pots, grouped by host-plant genotype and *Rhinanthus* treatment. The number of aphids on each plant in the HD is shown relative to the mean number of aphids per plant from the LD pots. When the value is positive, it shows that there were more aphids per plant in the HD pots, and when the value is negative, there were more aphids per plant in the LD pots. Smaller black stars indicate where the aphid number per plant for HD pots was significantly different (*P *<* *0.05) from the mean number per plant in the LD pots. Larger red stars indicate where the aphids are showing active choice toward or away from particular plant genotypes, determined by comparing the relative number of aphids in HD and LD pots to the average performance of the aphid on these plant genotypes from Fig. 2 data. Error bars are ±1 SE.

At the pot level, there was no effect of plant diversity or *Rhinanthus* treatment on the number of aphids on the plants, but diversity was a significant effect on aphid clustering (Table 1). As the number of aphids in the pots did not differ, the effects are due to the numbers of aphids on each plant within a pot. By comparing the number of aphids per plant in the HD pots with the average number per plant in the LD pots, inferences on the relative effects of preference and performance can be made (Fig. 5). The relative number of aphids differed among *Rhinanthus* treatment, depending on aphid genotype (green aphids: *Χ*^2^ = 14.31, df = 2, *P *<* *0.001; brown aphids: *Χ*^2^ = 15.12, df = 2, *P *<* *0.001) and also on barley genotype (green aphids by a barley genotype x aphid genotype interaction *Χ*^2^ = 12.99, df = 5, *P *=* *0.023; brown aphids by just a barley genotype main effect *Χ*^2^ = 84.5, df = 5, *P *<* *0.001). When these results are compared with aphid performance on each plant (Fig. 2), there are four particular aphid-plant genotype combinations that indicate aphid active choice is occurring (Fig. 5). CLO7 aphids show a reduced performance on Steptoe (Fig. 2) but are actually found in greater numbers on this host-plant genotype in the HD pots (Fig. 5), indicating active movement of aphids to this host plant. Active choice was also shown by DAV95 aphids for Morex (Figs. 2 and 5). Both HF92a and DAV95 aphids exhibit a high reproductive performance on the Kym host-plant genotype (Fig. 2) but are found less often on this plant genotype in HD pots, showing active choice away from this host plant (Fig. 5). However, this active choice for HF92a aphids against Kym is only seen for the Inverness *Rhinanthus* treatment, showing that *Rhinanthus* also mediates these interactions.

## Discussion

In this paper, we show that the distribution of aphids on host plants is influenced by direct and indirect genetic interactions among the members of a multispecies community (biotic environment). The system consisted of a host plant (*H. vulgare*; six genotypes), a hemi-parasitic plant (*Rhinanthus*; two populations), and aphids (*S. avenae*; four genotypes from two color morphs). The aphids showed greater clustering in pots with six different host-plant genotypes (high genotypic diversity; HD) than those with six plants from only one genotype (low genotypic diversity; LD). While there was no effect of plant genotype diversity on aphid number, plant genotypic diversity explained up to 24% of the variation in aphid clustering among treatments. We also considered the two host-plant diversity levels separately to further explore multitrophic interactions in these communities. Within both the diversity levels, a four-way interaction among the specific genotype of the host plant, the *Rhinanthus* treatment, and the genotypic identity of the aphids (own and interacting aphid) influenced the distribution of the brown aphids across the host plants, whereas the green aphids were only influenced by their own genotype and the genotype of the host plant. A comparison of the relative numbers of aphids among the host-plant genotypes in the HD and LD indicated there was active choice both toward and away from particular host-plant genotypes, which was mediated by the focal aphid genotype and *Rhinanthus* treatment.

The higher-order interaction term influencing the spatial distribution of the brown aphids means that all members of the community were important. While the two diversity levels showed similar results, the mechanisms driving them are likely to be quite different as there are a number of potential effects present in the HD pots that do not occur in the LD pots. These include aphid preference for different host-plant genotypes (Zytynska and Preziosi 2011, 2013); differential *Rhinanthus* performance (Rowntree et al. 2011a) on different host-plant genotypes; and intraspecific interactions between the host-plant genotypes (Donald 1951). The interaction between *Rhinanthus* and the aphids is assumed to have occurred indirectly, via the host plant, as we observed no aphids on the *Rhinanthus* itself throughout the experiment. *Rhinanthus* plants parasitize the roots of the host plants, gaining nutrients via the xylem (Seel and Jeschke 1999) and the aphids feed on the host-plant phloem-sap preferring to colonize the leaves and flowers of barley (Dent 2000). The relationship between *Rhinanthus* and aphids might be assumed as antagonistic as they compete for plant nutrients; however, work by Ewald et al. (2011) on the same aphid and hemi-parasitic plant species, but a different host-plant species (*Holcus lanatus*), showed the aphids had preference for, and increased population growth on, the grass when it was parasitized by *R. minor*. This may be driven by plant-induced defenses where infestation by the parasitic plant reduces the production of antiherbivore defenses, as has been demonstrated in a tomato-army worm-parasitic plant system (Runyon et al. 2008). Host-plant genetic variation can also influence tolerance to infection by *R. minor* (Rowntree et al. 2011a); thus, some parasitized host-plant genotypes could still provide a good environment for aphids while others do not. In the current study, DAV95 aphids on Morex show a generally high reproductive performance but exhibit greater active choice for this host-plant genotype when there is Somerset *Rhinanthus* present. A possible explanation for these results is that the Somerset *Rhinanthus* may avoid parasitizing the Morex, and it is therefore free from infection and presents a higher quality environment for the aphids. However, we would then expect to see similar numbers of aphids when there is no *Rhinanthus*. Alternatively, the Somerset *Rhinanthus* may preferentially attach to Morex and through facilitation create a better environment for the aphids (Ewald et al. 2011). Although we cannot show whether the *Rhinanthus* preferentially attached to particular host-plant genotypes from our data, we show that variation among the *Rhinanthus* populations (Inverness and Somerset) can alter plant-aphid genetic interactions and change the distribution of aphids across host-plants.

The final aspect of the community was the effect of the interacting aphid genotypes (intraspecific interaction), which is considered to occur via the plant as no physical fighting has ever been observed with these aphids and they co-exist on the plants. As aphids feed on a plant, they induce the expression of defense-related genes (Smith and Boyko 2006), and this is a potential mechanism for the effect of an interacting aphid on host preference and performance of a competitor aphid. In this case, the presence of one aphid genotype creates an environment that is not tolerated by another aphid genotype, causing it to move away or reducing the reproductive rate (Zytynska and Preziosi 2013). Through this mechanism, we might see the resulting differences in aphid spatial distribution as shown in this current study. As the interactions become more complex (i.e., with different interacting aphid genotypes in a high-diversity plant community), the preference for particular host-plant genotypes changes depending on the identity of those you interact with. This is an example of multiple IIGE's operating at the same time on the focal individual and is an explanation for the lack of correlation previously seen regarding the choice of herbivores to those plants that infer the highest fitness in a performance assay (Thompson 1988; McCauley et al. 1990).

Genetic diversity is the basis of evolutionary change in a population and IGEs (intraspecific)/IIGEs (interspecific) can both promote and hinder evolution in a species depending on the interaction type (Bailey 2012). These interactions can also have strong ecological consequences (Wolf et al. 1998; Whitham et al. 2006; Hughes et al. 2008; Rowntree et al. 2011b), for example, through fitness or performance effects acting between the interacting members in a community on either the same or different trophic levels (Hughes et al. 2008). Here, we show that genetic interactions among species influence the spatial distribution of herbivores in a population through changes in active choice of the aphids rather than purely through differential reproductive performance. Indeed, if an aphid chooses the host plant that infers the highest fitness then the effects of performance and preference will strongly increase aphid numbers on this plant; however, these interactions may also provide a mechanism to regulate aphid population sizes across host plants if aphids actively choose those plants that infer a lower fitness and move away from those inferring high fitness. Movement of herbivores to preferred plants in diverse patches may also feedback to enhance the plant fitness (Johnson et al. 2006) and increase food web complexity (Bukovinszky et al. 2008).

In conclusion, we have shown that the distribution of aphids on a population of host plants, but not the abundance, is influenced by both direct and indirect biotic interactions with other members of the community. We found that even in a highly diverse community direct and indirect species interactions have the potential to significantly alter the distribution of aphids among the host plants. Although genetic diversity is not always important for ecological processes (Hughes et al. 2008), when there is genetic variation for important ecological traits that influence species interactions, such variation can have strong ecological consequences. It now remains to be seen whether these results from model systems translate to ecologically important effects in natural systems and in which situations genetic diversity is important for community and ecological processes.

## Supporting Information

**Table S1.** Full statistical results for all pots (using sum per pot). **Table S2.** Full statistical results for low-diversity pots. **Table S3.** Full statistical results for high-diversity pots.
