# Supplementary material for: Host-plant genotypic diversity and community genetic interactions mediate aphid spatial distribution
Source: Ecol Evol. 2013 Dec 15;4(2):121–31. doi: 10.1002/ece3.916 (PMC3925376; doi:10.1002/ece3.916)
Supplement: Supplementary file 1 — Table S1. Full statistical results for all pots (using sum per pot). Table S2. Full statistical results for low-diversity pots. Table S3. Full statistical results for high-diversity pots. [file ece30004-0121-sd1.docx]

Table S1 Full statistical results for all pots (using sum per pot)

| **ALL APHIDS BY POT** | **Total aphid number** | | | **Clustering of aphids** | | |
| --- | --- | --- | --- | --- | --- | --- |
|  | **df** | **F** | **P** | **df** | **F** | **P** |
| **Biomass (covariate)** | 1,252 | 2.34 | 0.127 | **1,272** | **4.94** | **0.027** |
| **Aphid pair** | **3,273** | **9.84** | **<0.001** | 3,269 | 1.52 | 0.210 |
| **Diversity** | 1,272 | 3.23 | 0.069 | **1,272** | **50.74** | **<0.001** |
| **Rhinanthus** | 2,270 | 1.06 | 0.347 | **2,272** | **4.33** | **0.014** |
| Rhinanthus x Aphid pair | 6,264 | 2.00 | 0.066 | 6,258 | 0.72 | 0.631 |
| Diversity x Rhinanthus | 4,262 | 1.50 | 0.226 | 2,264 | 0.64 | 0.315 |
| Diversity x Aphid pair | 3,259 | 1.04 | 0.373 | 3,266 | 2.35 | 0.073 |
| Div x Rhin x Aphid pair | 6, 253 | 1.10 | 0.365 | 6,252 | 0.17 | 0.986 |

| **APHIDS BY POT** | **Green Number** | | | **Green clustering** | | | **Brown Number** | | | **Brown clustering** | | |
| --- | --- | --- | --- | --- | --- | --- | --- | --- | --- | --- | --- | --- |
|  | **df** | **F** | **P** | **df** | **F** | **P** | **df** | **F** | **P** | **df** | **F** | **P** |
| Biomass (covariate) | 2,252 | 0.03 | 0.859 | 1,252 | 1.98 | 0.161 | **1,274** | **4.70** | **0.031** | 1,274 | 4.69 | 0.031 |
| **Diversity** | 1,273 | 2.96 | 0.086 | **1,274** | **47.65** | **<0.001** | 1,273 | 1.86 | 0.173 | **1,274** | **27.45** | **<0.001** |
| **Green Aphid** | **1,274** | **12.98** | **<0.001** | **1,274** | **6.38** | **0.012** | 1,272 | 0.19 | 0.669 | 1,272 | 0.26 | 0.613 |
| Rhinanthus | 2,271 | 0.37 | 0.691 | 2,272 | 2.66 | 0.072 | 2,270 | 0.19 | 0.823 | 1,270 | 0.38 | 0.683 |
| **Brown Aphid** | **1,274** | **4.24** | **0.041** | 1,271 | 0.40 | 0.527 | **1,274** | **34.90** | **<0.001** | 1,273 | 1.87 | 0.172 |
| Diversity x Rhinanthus | 2,269 | 0.42 | 0.656 | 2,268 | 0.13 | 0.877 | 2,268 | 1.92 | 0.149 | 2,268 | 1.54 | 0.217 |
| Diversity x Green Aphid | 1,268 | 0.48 | 0.489 | 1,270 | 3.58 | 0.060 | 1,267 | 1.19 | 0.278 | 1,267 | 0.10 | 0.759 |
| Diversity x Brown Aphid | 1,267 | 1.21 | 0.273 | 1,267 | 1.70 | 0.193 | 1,266 | 0.11 | 0.738 | 1,266 | 1.96 | 0.163 |
| Rhinanthus x Green Aphid | 2,265 | 1.68 | 0.187 | 2,265 | 0.47 | 0.231 | 2,264 | 0.55 | 0.580 | 2,264 | 0.51 | 0.603 |
| Rhinanthus x Brown Aphid | 2,263 | 2.23 | 0.109 | 2,263 | 0.23 | 0.795 | 2,262 | 2.54 | 0.081 | 2,262 | 1.42 | 0.244 |
| Green x Brown Aphid | 1,262 | 0.20 | 0.656 | 1,262 | 0.13 | 0.718 | 1,261 | 0.00 | 0.993 | 1,261 | 1.31 | 0.253 |
| Diversity x Rhin x Green | 2,260 | 1.92 | 0.148 | 2,260 | 0.09 | 0.915 | 2,259 | 0.41 | 0.664 | 2,259 | 0.75 | 0.474 |
| Diversity x Rhin x Brown | 2,258 | 0.19 | 0.825 | 2,258 | 0.76 | 0.468 | 2,257 | 2.41 | 0.092 | 2,257 | 0.52 | 0.593 |
| Diversity x Green x Brown | 1,257 | 0.78 | 0.379 | 1,257 | 0.44 | 0.507 | 1,256 | 0.94 | 0.334 | 1,256 | 1.06 | 0.305 |
| Rhin x Green x Brown | 2,255 | 2.28 | 0.104 | 2,255 | 0.92 | 0.401 | 2,254 | 0.69 | 0.501 | 2,254 | 0.48 | 0.621 |
| Div x Rhin x Green x Brown | 2,253 | 0.09 | 0.912 | 2,253 | 1.21 | 0.300 | 2,252 | 0.19 | 0.824 | 2,252 | 0.79 | 0.455 |

*Notes: Bold indicated the terms remained in the minimal adequate model*

Table S2 Full statistical results for low diversity pots

| **LOW DIVERSITY** |  | **Number of green aphids** | | **Clustering of green aphids** | | **Number of brown aphids** | | **Clustering of brown aphids** | |
| --- | --- | --- | --- | --- | --- | --- | --- | --- | --- |
|  | **df** | **Chi-sq** | **P** | **Chi-sq** | **P** | **Chi-sq** | **P** | **Chi-sq** | **P** |
| **Host-plant genotype** | **5** | **35.51** | **<0.001** | **20.34** | **0.001** | **23.18** | **<0.001** | **16.60** | **0.005** |
| Rhinanthus | **2** | **0.43** | **0.806** | 1.33 | 0.515 | **3.48** | **0.175** | **2.82** | **0.245** |
| **Green Aphid** | **1** | **0.01** | **0.999** | 0.01 | 0.999 | **0.80** | **0.370** | **0.328** | **0.567** |
| Brown Aphid | **1** | **2.43** | **0.119** | 0.01 | 0.999 | **18.47** | **<0.001** | **0.01** | **0.999** |
| Genotype x Rhinanthus | **10** | **6.26** | **0.793** | 10.46 | 0.401 | **20.42** | **0.026** | **10.80** | **0.374** |
| Genotype x Green Aphid | **5** | **16.27** | **0.006** | 1.80 | 0.876 | **3.14** | **0.680** | **1.68** | **0.891** |
| Genotype x Brown Aphid | **5** | **4.14** | **0.529** | 3.08 | 0.687 | **5.44** | **0.364** | **1.92** | **0.870** |
| Rhinanthus x Green Aphid | **2** | **3.61** | **0.164** | 1.13 | 0.567 | **1.62** | **0.445** | **0.48** | **0.785** |
| Rhinanthus x Brown Aphid | **2** | **4.06** | **0.131** | 1.04 | 0.593 | **11.78** | **0.003** | **2.21** | **0.331** |
| Green x Brown Aphid | **1** | **0.30** | **0.583** | 1.03 | 0.311 | **0.01** | **0.999** | **1.94** | **0.163** |
| Genotype x Rhin x Green | **10** | **11.52** | **0.319** | 4.86 | 0.900 | **8.78** | **0.553** | **3.47** | **0.968** |
| Genotype x Rhin x Brown | **10** | **6.75** | **0.748** | 8.73 | 0.558 | **0.93** | **0.999** | **7.73** | **0.656** |
| Genotype x Green x Brown | **5** | **4.41** | **0.491** | 7.30 | 0.199 | **6.22** | **0.286** | **3.62** | **0.605** |
| Rhin x Green x Brown | **2** | **4.22** | **0.121** | 1.84 | 0.399 | **0.74** | **0.691** | **3.36** | **0.186** |
| Gen x Rhin x Green x Brown | **10** | **32.47** | **<0.001** | 1.18 | 0.999 | **20.23** | **0.027** | **25.19** | **0.005** |

*Notes: Bold indicated the terms remained in the minimal adequate model, number of aphids was natural-log transformed for better model fit*

Table S3 Full statistical results for high diversity pots

| **HIGH DIVERSITY** |  | **Number of green aphids** | | **Clustering of green aphids** | | **Number of brown aphids** | | **Clustering of brown aphids** | |
| --- | --- | --- | --- | --- | --- | --- | --- | --- | --- |
|  | **df** | **Chi-sq** | **P** | **Chi-sq** | **P** | **Chi-sq** | **P** | **Chi-sq** | **P** |
| **Host-plant genotype** | **5** | **219.49** | **<0.001** | **66.35** | **<0.001** | **136.55** | **<0.001** | **18.35** | **0.002** |
| Rhinanthus | 2 | 1.53 | 0.465 | 1.90 | 0.387 | **0.29** | **0.867** | **1.95** | **0.378** |
| **Green Aphid** | 1 | 1.68 | 0.193 | **5.75** | **0.016** | **0.11** | **0.742** | **0.00** | **0.999** |
| Brown Aphid | 1 | 0.91 | 0.340 | 1.90 | 0.168 | **18.56** | **<0.001** | **2.53** | **0.112** |
| Genotype x Rhinanthus | 10 | 14.71 | 0.143 | 9.55 | 0.481 | **13.80** | **0.183** | **7.87** | **0.642** |
| Genotype x Green Aphid | 5 | 7.62 | 0.179 | 6.83 | 0.233 | **2.93** | **0.710** | **3.36** | **0.645** |
| Genotype x Brown Aphid | 5 | 6.52 | 0.259 | 4.88 | 0.431 | **1.21** | **0.944** | **0.01** | **0.999** |
| Rhinanthus x Green Aphid | 2 | 4.92 | 0.085 | 1.32 | 0.517 | **2.63** | **0.269** | **12.92** | **0.002** |
| Rhinanthus x Brown Aphid | 2 | 3.13 | 0.209 | 0.26 | 0.877 | **0.16** | **0.924** | **0.85** | **0.654** |
| Green x Brown Aphid | 1 | 0.03 | 0.873 | 0.02 | 0.879 | **1.29** | **0.257** | **0.08** | **0.782** |
| Genotype x Rhin x Green | 10 | 14.26 | 0.162 | 6.86 | 0.739 | **9.61** | **0.475** | **10.88** | **0.367** |
| Genotype x Rhin x Brown | 10 | 9.38 | 0.496 | 4.41 | 0.927 | **7.29** | **0.698** | **11.66** | **0.309** |
| Genotype x Green x Brown | 5 | 6.99 | 0.222 | 1.32 | 0.932 | **2.29** | **0.808** | **5.31** | **0.379** |
| Rhin x Green x Brown | 2 | 2.64 | 0.267 | 1.13 | 0.567 | **0.13** | **0.938** | **0.21** | **0.902** |
| Gen x Rhin x Green x Brown | 10 | 9.85 | 0.454 | 11.32 | 0.333 | **18.27** | **0.051** | **22.72** | **0.012** |

*Notes: Bold indicated the terms remained in the minimal adequate model, number of aphids was natural-log transformed for better model fit*
